# Combined Metabolomic and Transcriptomic Analysis Reveals Allantoin Enhances Drought Tolerance in Rice

**DOI:** 10.3390/ijms232214172

**Published:** 2022-11-16

**Authors:** Shuai Lu, Zichang Jia, Xiangfeng Meng, Yaoyu Chen, Surong Wang, Chaozhen Fu, Lei Yang, Rong Zhou, Baohua Wang, Yunying Cao

**Affiliations:** 1 School of Life Sciences, Nantong University, Nantong 226019, China; 2 State Key Laboratory Breeding Base of Green Pesticide and Agricultural Bioengineering, Key Laboratory of Green Pesticide and Agricultural Bioengineering, Ministry of Education, Center for Research and Development of Fine Chemicals, Guizhou University, Guiyang 550025, China

**Keywords:** transcriptome, untargeted metabolome, purine metabolism, allantoin, ROS, rice

## Abstract

Drought is a misfortune for agriculture and human beings. The annual crop yield reduction caused by drought exceeds the sum of all pathogens. As one of the gatekeepers of China’s “granary”, rice is the most important to reveal the key drought tolerance factors in rice. Rice seedlings of Nipponbare (*Oryza sativa* L. ssp. *Japonica*) were subjected to simulated drought stress, and their root systems were analyzed for the non-targeted metabolome and strand-specific transcriptome. We found that both DEGs and metabolites were enriched in purine metabolism, and allantoin accumulated significantly in roots under drought stress. However, few studies on drought tolerance of exogenous allantoin in rice have been reported. We aimed to further determine whether allantoin can improve the drought tolerance of rice. Under the treatment of exogenous allantoin at different concentrations, the drought resistant metabolites of plants accumulated significantly, including proline and soluble sugar, and reactive oxygen species (ROS) decreased and reached a significant level in 100 μmol L^−1^. To this end, a follow-up study was identified in 100 μmol L^−1^ exogenous allantoin and found that exogenous allantoin improved the drought resistance of rice. At the gene level, under allantoin drought treatment, we found that genes of scavenge reactive oxygen species were significantly expressed, including peroxidase (POD), catalase (CATA), ascorbate peroxidase 8 (APX8) and respiratory burst oxidase homolog protein F (RbohF). This indicates that plants treated by allantoin have better ability to scavenge reactive oxygen species to resist drought. Alternative splicing analysis revealed a total of 427 differentially expressed alternative splicing events across 320 genes. The analysis of splicing factors showed that gene alternative splicing could be divided into many different subgroups and play a regulatory role in many aspects. Through further analysis, we restated the key genes and enzymes in the allantoin synthesis and catabolism pathway, and found that the expression of synthetase and hydrolase showed a downward trend. The pathway of uric acid to allantoin is completed by uric acid oxidase (UOX). To find out the key transcription factors that regulate the expression of this gene, we identified two highly related transcription factors *OsERF059* and *ONAC007* through correlation analysis. They may be the key for allantoin to enhance the drought resistance of rice.

## 1. Introduction

Drought is one of the major natural disasters in China and the most serious meteorological disaster. Drought is a long-term climatic disaster, abnormal atmospheric circulation is the direct cause of drought, and climate warming and drying are important reasons for the frequent occurrence of drought [1]. When the temperature rises, the surface evaporation increases, the natural precipitation decreases, and the soil moisture drop rapidly, resulting in the simultaneous occurrence of atmospheric drought and soil drought, thus causing serious damage to agricultural production [2]. By 2050, the food crops we produce today, particularly rice, wheat, soybeans, and corn, will need to increase by 87% to meet the needs of the world’s growing population. Therefore, drought is a threat to world food security [3]. More than half of the world’s population uses rice as the main source of carbohydrates [4]. Rice is a water-intensive crop and irrigated rice accounts for 53% of the global rice cultivation area. The availability and availability of freshwater determine global rice production [5]. As human populations grow and water resources become depleted, developing drought-resistant crops is critical to preventing crop yield losses from drought stress [6].

Drought makes it difficult for plants to obtain water and nutrients, leading to cell death [7]. Studies have shown that the damage of secondary oxidative stress to plants cannot be ignored [8]. Drought-induced oxidative stress is due to the production of reactive oxygen species (ROS), such as superoxide anion (O_2_^·−^), singlet oxygen (^1^O_2_), hydroxyl radical (OH^−^), and hydrogen peroxide (H_2_O_2_) [9,10]. These ROS are highly cytotoxic and can react with important biomolecules such as lipid, protein, and nucleic acid, causing lipid peroxidation, protein denaturation, and DNA mutation, respectively [11]. The major organelles of ROS generation in cells are chloroplasts, mitochondria, peroxisomes, apoplast, and endoplasmic reticulum [12]. There are two major avenues to detoxify ROS in plants: enzymatic and nonenzymatic scavenging mechanisms. Nonenzymatic antioxidants include low-molecular-weight antioxidants such as α-tocopherol, carotenoids, ascorbic acid (AsA), reduced glutathione (GSH), flavonoids, and total phenols. Additional nonenzymatic antioxidants, such as soluble sugar, trehalose, polyamines, proline, and glycine betaine, also contribute to the regulation of ROS. Among them, proline not only regulates permeability in adversity but also inhibits ROS, stabilizing ROS scavenging enzyme and protects plant tissues from ROS damage, glycine betaine mainly accumulates in chloroplasts and protects photosynthetic apparatus during oxidative stress [13]. Major ROS-scavenging enzymes are superoxide dismutase (SOD), peroxidase (POD), catalase (CAT), ascorbate peroxidase (APX), glutathione peroxidase (GPX), and glutathione reductase (GR) [14,15].

Allantoin (N-(2,5-Dioxo-4-imidazolidinyl) urea) is an important member of the plant nitrogen cycle system. Since Macalister [16] (1912) first extracted allantoin from *Symphytum officinale* at the beginning of the last century, allantoin has been considered an intermediate metabolite in the process of purine decomposition in plants, participating in nitrogen transport, storage, and reuse in plants [17,18,19]. In addition, early studies showed that exogenous application of allantoin can improve the yield of crops [20]. In recent years, studies have found that plants are accompanied by allantoin accumulation under such stress conditions as pathogen infection [21], low temperature [22,23], nutrient deficiency [24], dark treatment [25], high salt [26,27], and drought [28,29]. At the same time, higher allantoin levels of in vivo or exogenous allantoin can induce a series of stress responses in plants [30]. Analysis of allantoin level in rice grains found that bran always had the highest allantoin level, followed by brown rice and milled rice [31]. Allantoin accumulation through overexpression of ureide permease1 improves rice growth under limited nitrogen conditions [32]. Allantoin accumulates under long-term salt stress. Adjustment of nitrogen metabolism in rice roots is likely to be closely related to maintain the growth potential and increase the stress tolerance [33]. Studies have shown that enhancing the activity of catalysis of xanthine dehydrogenase (XDH) can regulate the synthesis of urea-related substances, improve plant the antioxidant capacity, effectively delay the aging process in rice leaves, and increase rice yield [34]. These results suggest that allantoin plays an important role in the growth and development of rice. At present, the application of exogenous allantoin in rice drought resistance has not been systematically studied, and the specific molecular mechanism has yet to be revealed.

We aimed to further determine how the enzymatic and non-enzymatic reactions of the roots function in the face of drought stress in rice. We performed transcriptomic and untargeted metabolomic profiling of rice roots under drought stress. Interestingly, we found that in the purine metabolism pathway, allantoin was accumulated under drought stress, and genes related to allantoin metabolism were down-regulated. We also found that the drought tolerance of rice was enhanced under exogenous allantoin treatment, and the expression of some genes related to scavenging reactive oxygen species was up-regulated, indicating that it can improve the drought resistance of rice by increasing the scavenging ability of rice to reactive oxygen species. We further speculated that *OsERF059* or *ONAC007* may be the key transcription factor regulating the allantoin synthesis gene *OsUOX*.

## 2. Results

### 2.1. Identification of Core Genes and Metabolites in Rice under Drought Stress

**Figure 1:**
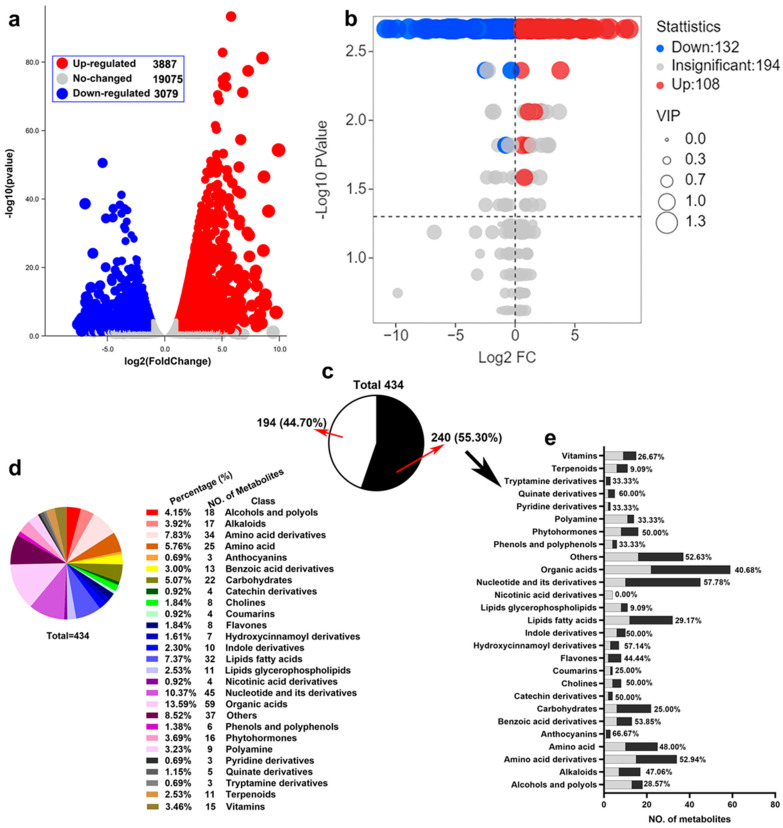
Summary of changes in transcript and metabolite abundance in rice in response to drought stress. (**a**) Volcano map of transcriptome genes under drought stress. **(b**) Volcano map of metabolome metabolites under drought stress. (**c**) All metabolites detected by the metabolome were classified. Black was the proportion of the number of differential metabolites, and white was the non-differential part. (**d**) Classification of 434 metabolites and their proportions. (**e**) The amount and proportion of 240 different metabolites in various metabolites and their derivatives were described in detail.

To fully understand the changes in transcription and metabolism in rice roots under drought stress, widely targeted metabolomics and RNA-Seq analysis on rice roots treated with 20% PEG6000 for 5 days were performed. For RNA SEQ, three independent replicates per treatment and a total of 6 libraries were sequenced. For each sample, the generated high-quality clean reads were mapped onto the annotated transcripts in the Nipponbare reference genome IRGSP-1.0. To identify genes differentially expressed under drought treatment, paired differential expression profiling (FDR ≤ 0.05, absolute fold change ≥ 2.0) was performed between the treatment group and the control group. DEG volcano analysis showed that 3887 genes were up-regulated and 3079 genes were down-regulated (Figure 1a). For metabolite profiling, each treatment was repeated 6 times, and a total of 12 samples were obtained. Among them, we identified 434 metabolites, of which 108 were up-regulated and 132 were down-regulated (Figure 1b). To further identify the categories of metabolites (Figure 1c), we classified and counted them and found that among the 434 metabolites, organic acids accounted for 13.59%, followed by nucleotides and their derivatives at 10.37% (Figure 1d). We further classified the differential metabolites statistically and found that the differential metabolites were mainly organic acids, nucleotides and their derivatives, amino acids, and their derivatives, and fatty acids and carbohydrates accounted for a large proportion (Figure 1e). They all belong to the basic metabolites of plant cells.

### 2.2. Enrichment Analysis of DEGs and DEMs in Transcriptome and Metabolome Expression Profiles

**Figure 2:**
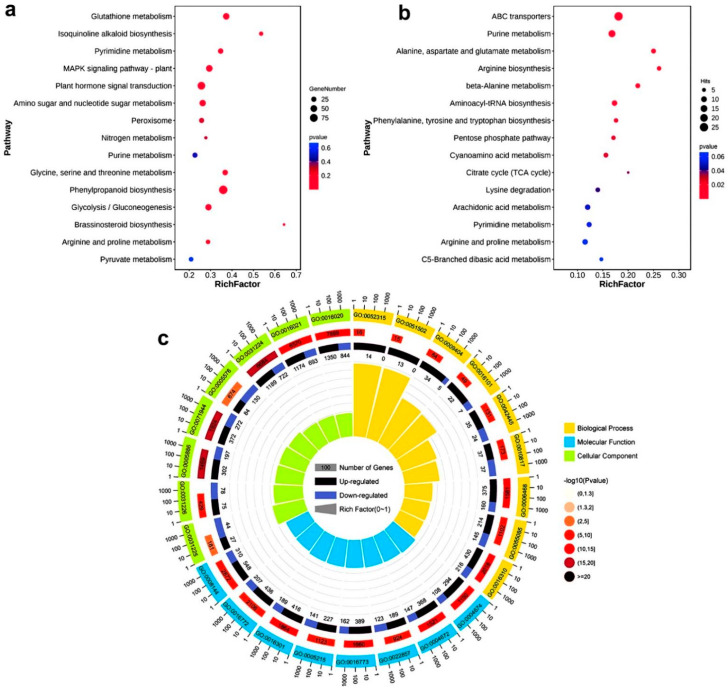
KEGG analysis and GO analysis of transcriptome DEGs under drought stress and KEGG analysis of DEMs. (**a**) KEGG analysis of transcriptome DEGs, only 15 items are shown. (**b**) KEGG analysis of metabolome DEMs, only 15 items are shown. (**c**) Go enrichment analysis of transcriptome DEGs, functions corresponding to specific items, and DEGs are shown in Table S1.

To clarify the possible functions of the DEGs and DEMs, KEGG (Kyoto Encyclopedia of Genes and Genome) enrichment analysis showed that the DEGs were mainly enriched in glutathione metadata, isoquinoline alkaloid, biosynthesis, pyrimidine metadata, MAPK signaling pathway-plant, plant hormone signal transduction, amino sugar, and nucleotide sugar metadata, peroxide, nitrogen metadata, purine metadata, glycine, serine and threonine metabolism, phenylpropanoid biosynthesis, glycolysis/gluconeogenesis, brassinosteroid biosynthesis, arginine and proline metabolism, and pyruvate metabolism, etc. (Figure 2a). KEGG results of DEMs showed that they were mainly enriched in ABC transporters, purine metabolism, alanine, aspartate and glutamate metabolism, arginine biosynthesis, beta-Alanine metabolism, aminoacyl-tRNA biosynthesis, phenylalanine, tyrosine, and tryptophan biosynthesis, pentose phosphate pathway, cyanoamino acid metabolism, citrate cycle (TCA cycle), lysine degradation, arachidonic acid metabolism, pyrimidine metabolism, arginine, and proline metabolism and C5-Branched dibasic acid metabolism, etc. (Figure 2b). We continued to enrich DEGs by GO (Gene Ontology) and found that they were mainly related to membrane transport, hormones, and peroxisomes (Figure 2c). Some other entry information is shown in Table S1. Interestingly, we found that KEGG analysis of DEGs and metabolites was enriched in the purine metabolic pathway, and a large amount of H_2_O_2_ or O_2_^−^ would be produced in the process of xanthine metabolism to form uric acid. The peroxide was also enriched in the analysis of the DEGs KEGG. Purine metabolism may be the key to rice response to drought stress by using enzymatic and non-enzymatic reactions.

### 2.3. Expression Profiles of Intermediates and Related Enzymes in the Allantoin Metabolism Pathway in Rice Roots under Drought Stress

**Figure 3:**
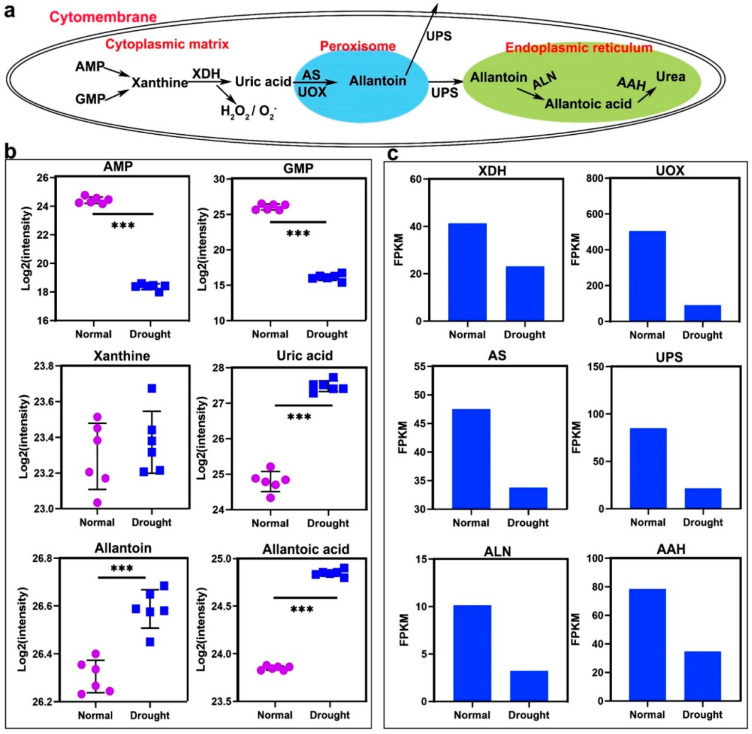
Synthesis and decomposition of allantoin in purine metabolism. (**a**) The relationship between the compounds, enzymes, and organelles involved in the synthesis, hydrolysis, and transport of allantoin in cells. (**b**) Under drought stress, the number of metabolites in the metabolic process of (**a**). (**c**) Under drought stress, the expression of enzymes in the metabolic process of (**a**). Data in (**b**) are presented as mean ± SD of three independent experiments, the differences between the drought treatment group and the control group are indicated by asterisks. Data in **c** are presented as mean ± SD of three independent experiments, and the differences between the drought treatment group and the control group are indicated by asterisks. Asterisks indicate significance (*** *p* < 0.001).

The main pathway of allantoin synthesis in higher plants is purine degradation (Figure 3a). After deamination, adenine monophosphate (AMP) and guanosine monophosphate (GMP) are both converted to xanthine, which in turn generates uric acid under the catalysis of XDH. Uric acid generates allantoin under the action of uric acid oxidase (UOX) and allantoin synthase (AS). Among them, the conversion of AMP and GMP to xanthine and the formation of uric acid are all completed in the cytoplasmic matrix [35], and the metabolism of uric acid to allantoin is carried out in the peroxisome [36]. Subsequently, allantoin can enter the cytoplasm under the action of ureide permease (UPS) to participate in a variety of metabolic activities [37], and can also be secreted to the extracellular matrix to participate in nitrogen transport [38], or expel plant bodies and play a role in allelopathy [39]. Allantoin catabolism mainly occurs in the endoplasmic reticulum [40]. First, allantoinase generates allantoic acid, and then it is converted into urea under the action of allantoic acid hydrolase (AAH). Under drought stress, the content of AMP and GMP decreased, the content of xanthine basically did not change, and the content of uric acid, allantoin, and allantoic acid increased significantly (Figure 3b). The detection peak of metabolites related to the allantoin metabolism pathway in positive and negative ion mode is shown in Figure S1. The related enzymes showed a downward trend (Figure 3c). Allantoin content in plants was positively correlated with most abiotic stress responses [41]. It is suggested that allantoin may play an important role in the drought resistance of rice.

### 2.4. Exogenous Allantoin Improves the Drought Resistance of Rice

**Figure 4:**
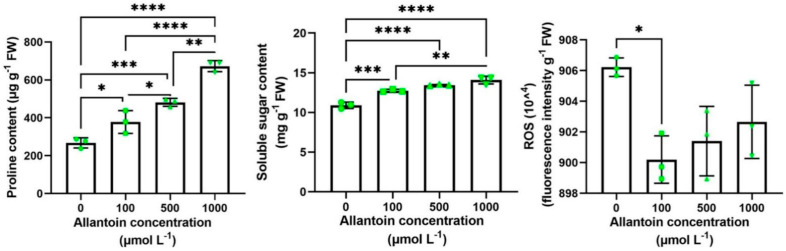
Accumulation of proline, soluble sugar and ROS in plants under drought stress. Rice seeds germinated at different exogenous allantoin concentrations for 22 days and were treated with 20% PEG 6000 to simulate drought. After 5 days, samples were taken to quantify proline, soluble sugar and ROS in plants. Data are expressed as the mean ± SD of three independent experiments. According to one-way ANOVA, significant differences between treatments are indicated by asterisks. Asterisks indicate significance (* *p* < 0.05, ** *p* < 0.01, *** *p* < 0.001, **** *p* < 0.0001).

**Figure 5:**
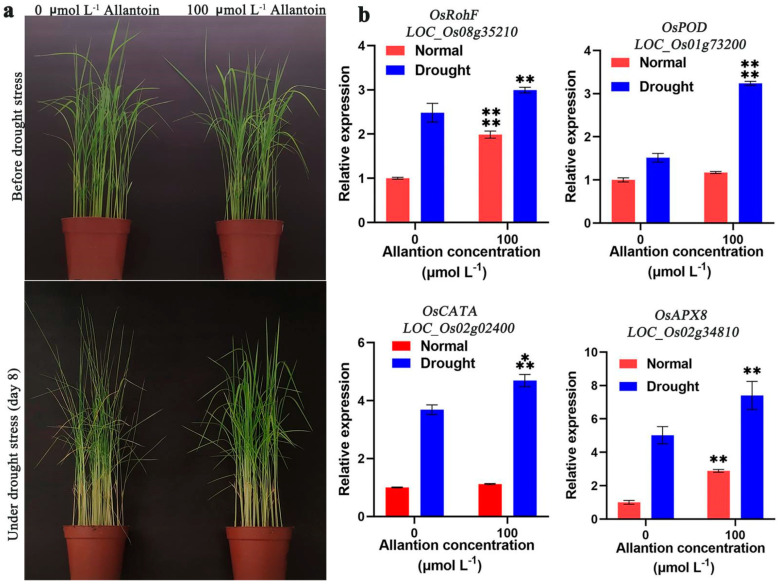
Allantoin improves the drought resistance in rice. (**a**) A total of 100 μmol L^−1^ allantoin treatment, the phenotype of rice after stopping watering. (**b**) Allantoin treatment of gene expression of ROS scavenging in plants under drought stress. Relative expression levels of the gene were normalized to the internal control of the *OsACTIN-1* (*LOC_Os05g36290*) gene. Data are expressed as the mean ± SD of three independent experiments. Asterisks indicate significance (** *p* < 0.01, *** *p* < 0.001, **** *p* < 0.0001).

To further determine whether exogenous allantoin can improve the drought resistance of rice, we selected a 20% PEG solution of allantoin with different concentration (0, 100, 500, 1000 μmol L^−1^) gradient to treat 3-week-old rice seedlings, and sampled them after 5 days of simulated drought to carry out quantitative experiments on proline, soluble sugar and ROS. The results showed that the proline content increased with the increase of allantoin concentration; the changing trend of soluble sugar was the same as that of proline; the content of ROS decreased, but the accumulation of ROS was the lowest at 100 μmol L^−1^ concentration and reached a significant difference level (Figure 4). Therefore, we set the concentration of exogenous allantoin to treat rice seedlings at 100 μmol L^−1^, and all subsequent experiments were conducted at this concentration. In a word, rice treated with different concentrations of allantoin has higher proline and soluble sugar in the face of drought and accumulates less reactive oxygen species, which indicates that exogenous allantoin treatment does affect the internal environment of rice so that it has a better internal environment to resist drought. By planting in soil culture, rice seedlings of about 3 weeks old showed better drought resistance in the allantoin treatment group after 8 days of water stoppage. The rice leaves of the control without allantoin group showed a large range of curls, while the allantoin treatment group only had a slight curl (Figure 5a). This indicated that exogenous allantoin could improve the drought tolerance of rice. The expression changes of some ROS-generating and scavenging-related genes, including RbohF (NADPH oxidase, *LOC_Os08g35210*), POD (peroxidase, *LOC_Os01g73200*), CATA (catalase, *LOC_Os02g02400*) and APX8 (ascorbate peroxidase, *LOC_Os02g34810*) [42] were analyzed (Figure 5b). RohF was induced to express about 1.9-fold under normal watering and exogenous allantoin treatment. Application of allantoin RohF also induced the expression of RohF of the drought treatment group, which increased by about 1.2 times. Rice seedlings with exogenous allantoin were not significantly different in POD expression compared to the control without adding allantoin under normal watering, but under drought stress, allantoin significantly induced the expression of POD, increasing by about 2.1 times. Similarly, the expression of CATA was not significantly different under normal watering, but allantoin significantly induced the expression of CATA folding, increasing by about 1.2 times under drought stress. The expression of APX8 in the treatment with allantoin increased about 2.4 times under normal watering, and APX8 was significantly induced by allantoin under drought stress, with an increase of about 1.6 times. These results indicate that exogenous allantoin enhances the drought resistance of rice by inducing the expression of ROS scavenging genes.

### 2.5. Identification of Key Transcription Factors Regulating Allantoin Metabolism

**Figure 6:**
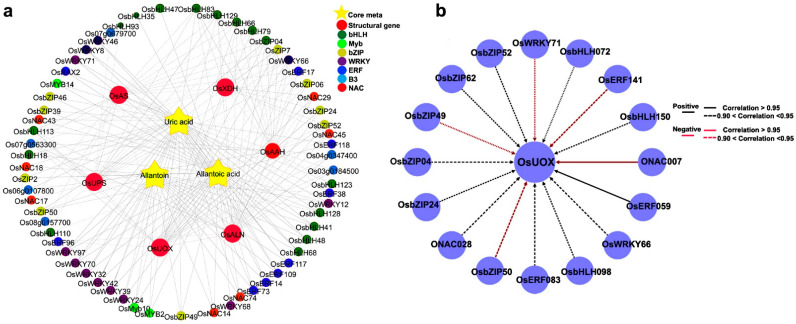
Metabolic regulatory network of allantoin. (**a**) Yellow stars represent key metabolites in the allantoin metabolic pathway, and large red circles represent structural genes involved in allantoin metabolism. Different colored circles represent different families of transcription factors identified in the same module. (**b**) Transcriptional regulatory network of *OsUOX.* The surrounding circles represent transcription factors identified as highly related to *OsUOX*.

To elucidate the regulatory role of key transcription factors that potentially control allantoin metabolism, we selected the regulatory network of allantoin metabolism, which is believed to improve the drought resistance of rice. Since the accumulation of allantoin under drought stress contributes to plant drought resistance, we focused on the analysis of the regulatory network of uric acid, allantoin, and allantoic acid genes, which play a key role in allantoin synthesis and metabolism. The six structural genes are involved in the synthesis, decomposition, and transport of allantoin. In the allantoin metabolic network, we identified 62 transcription factors highly related to the allantoin pathway (Figure 6a). UOX and AS genes were identified as related to allantoin synthesis. To characterize the key transcription factors that regulate the activation of structural genes encoding allantoin accumulation enzymes, we focused on the regulation of UOX, because this structural gene showed differential downregulation under drought stress. Regulatory network analysis showed that UOX transcripts were highly correlated with 16 transcription factors, including ERF, NAC, bZIP, WRKY, and bHLH families. We found that there were two transcription factors (OsERF059 and ONAC007) with high correlation (over 0.95) coefficients (Figure 6b). The typical regulatory motifs in the UOX promoter sequence were further searched and found that the UOX promoter sequence contains more ABRE motifs (Figure S2), which are abscisic acid response elements (Hwang et al., 2019). This motif can generally be found in the promoter region of many stress-induced genes. This may imply the important role of UOX under drought stress.

### 2.6. Differential Alternative Splicing Analysis

**Figure 7:**
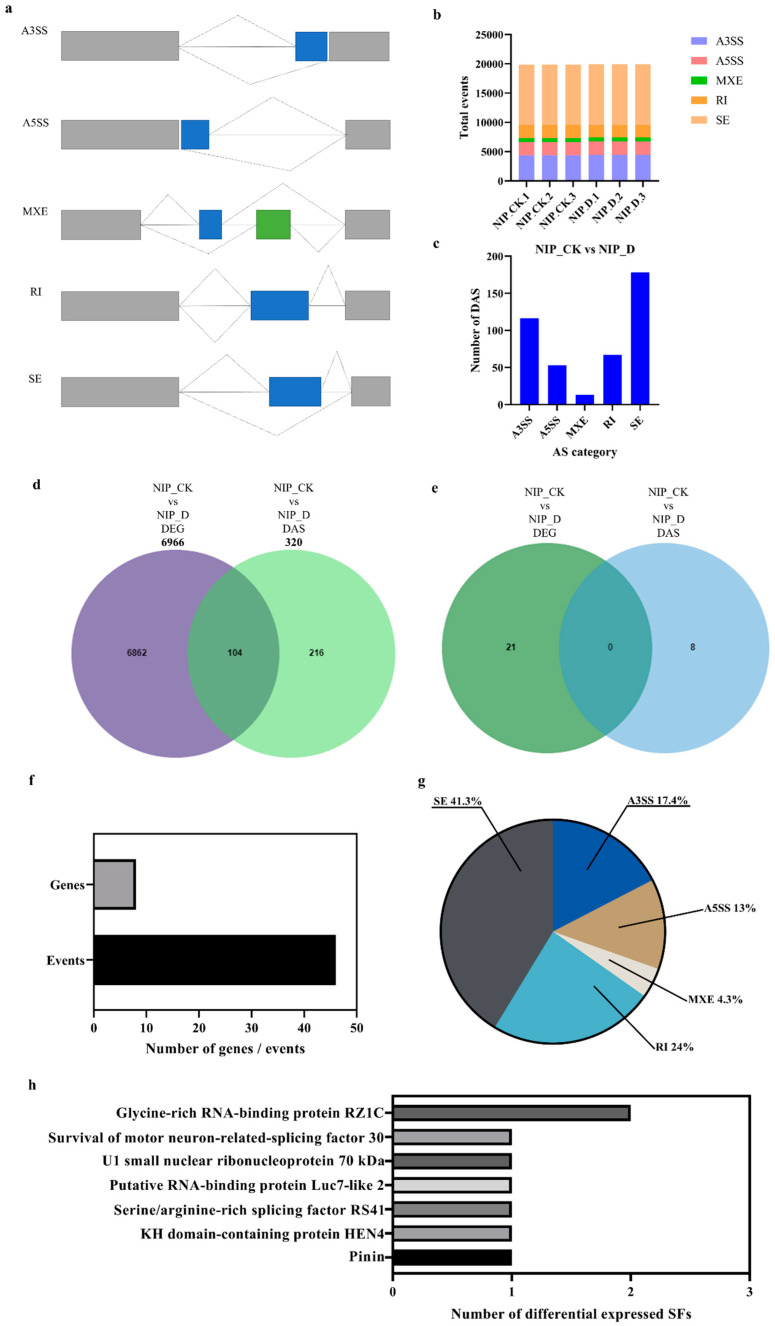
Identification and comparison of differentially expressed alternative splicing (DAS) and differentially expressed gene (DEG) datasets. (**a**) Main classification of alternative splicing. A3SS, alternative 3′ splice site; A5SS, alternative 5′ splice site; MXE, mutually exclusive exon; SE, skipped exon; RI, intron retention. (**b**) A summary of the number and types of identified AS. (**c**) DAS Events in NIP-CK vs. NIP-D comparison group. (**d**) Venn diagram represents the unique and shared genes between DEG and DAS datasets. Analysis of alternative splicing. (**e**) The Venn diagram represents identified splicing factors between DEG and DAS data sets. (**f**) Statistics of drought-affected DAS genes and events of splicing factors. (**g**) Pie chart distribution of the DAS events belonging to splicing factors. (**h**) Subgroup classification of splicing factors identified in DAS events.

RNA alternative splicing is an important gene expression regulation mechanism that is widely mediated by the spliceosome in eukaryotes [43]. Its existence increases the genetic diversity of proteins and provides more possibilities for the evolution of organisms [44]. Currently, known alternative splicing is mainly divided into five types: intron retention (RI), exon skipping (SE), alternative 5’ splice site (A5SS), alternative 3’ splice site (A3SS), and mutually exclusive exons (MXE) (Figure 7a) [45,46].

In this experimental study, for AS identification, more than 19,000 AS events were identified for each sample, among which SE and A3SS were the two most abundant AS events in all samples (Figure 7b). In both AS event types, A3SS leads to a variable 3’ untranslated end, and polyadenylation of the 3’ end of the transcript affects transcript localization and stability [47]. In addition, 427 DAS events in 320 genes were revealed in the examination of differential alternative splicing events in the NIP-CK vs. NIP-D comparison group. Figure 7c shows DAS events (FDR < 0.01) in the comparison groups of NIP-CK vs. NIP-D. When the DEG dataset was compared with the DAS dataset, only 104 genes out of 7182 genes were differentially regulated at the transcriptional and posttranscriptional levels (Figure 7d). These results suggest that AS may play an important and unique role in the drought tolerance of rice.

In addition, to further explore the mechanism of AS involvement in drought stress regulation, rice-related splicing factors were analyzed. A total of 21 genes were differentially expressed in the DEG dataset, and 46 AS events from eight splicing factor-related proteins were found in the DAS dataset (Figure 7e,f), whereas no AS events were found in the DEG dataset, suggesting that these splicing factors are specifically regulated by posttranscriptional mechanisms. Among the 46 splicing events, SE was the main splicing event, accounting for 41.3%, followed by RI, accounting for 24% (Figure 7g). The other three alternative splicing types, A3SS, A5SS and MXE, accounted for 34.7% of the total AS events (Figure 7g). According to the classification of the gene splicing database (SRGD http://www.plantgdb.org/SRGD/index.php, accessed on 21 September 2022), the DAS observation dataset of eight genes can be divided into seven subgroups (Figure 7h). The response to drought stress was carried out in various ways.

### 2.7. qPCR Validation of Possible Resistance Genes under Drought Stress

**Figure 8:**
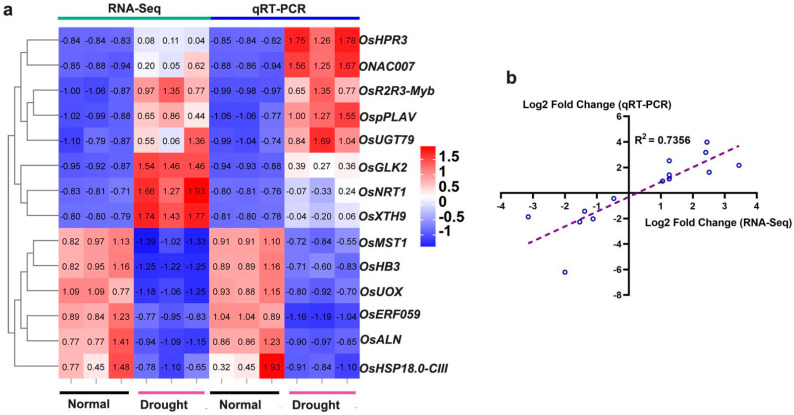
qRT-PCR confirmed the expression profiles obtained by RNA-Seq transcriptome analysis at different times after drought stress. (**a**) A total of 14 genes were selected from the DEGs for the mapping of expression profiles. (**b**) A linear graph of the correlation between the transcriptome and qRT-PCR was drawn. Relative expression levels of the gene were normalized to the internal control of the *OsACTIN-1* (*LOC_Os05g36290*) gene.

To further mine possible resistance genes, we validated transcriptome data under drought stress. Among them, we selected a total of 14 genes, of which eight were up-regulated and six were down-regulated, the upregulated genes include glyoxylate/hydroxypyruvate reduce (*OsHPR3*), transcription factor glk2 (*OsGLK2*), nitrate transporter (*OsNRT1*), UDP glucosyltransferase (*OsUGT79*), Patin-like phospholipase domain containing protein 2 (*OspPLAV*), xyloglucan endotransferase/hydrolase protein (*OsXTH9*), MYB related protein 306 and OsNAC7 protein. Down-regulated genes are allantoinase (*OsALN*), sugar transport protein MST1-like (*OsMST1*), 18.6 kDa class III heat shock protein-like (*OsHSP-18.0-CIII*), uricase (*OsUOX*), hemoglobin (*OsHB3*) and ethylene-responsive transcription factor (*OsERF059*). The expression pattern of genes related to allantoin metabolism under drought stress is consistent with the transcriptome (Figure 8), which further illustrates the important role of allantoin in drought resistance of rice. It contains two highly related transcription factors that may regulate OsUOX as previously analyzed. We performed correlation analysis on transcriptome and qPCR data, and the correlation coefficient was 0.7356, indicating the reliability of our data.

## 3. Discussion

**Figure 9:**
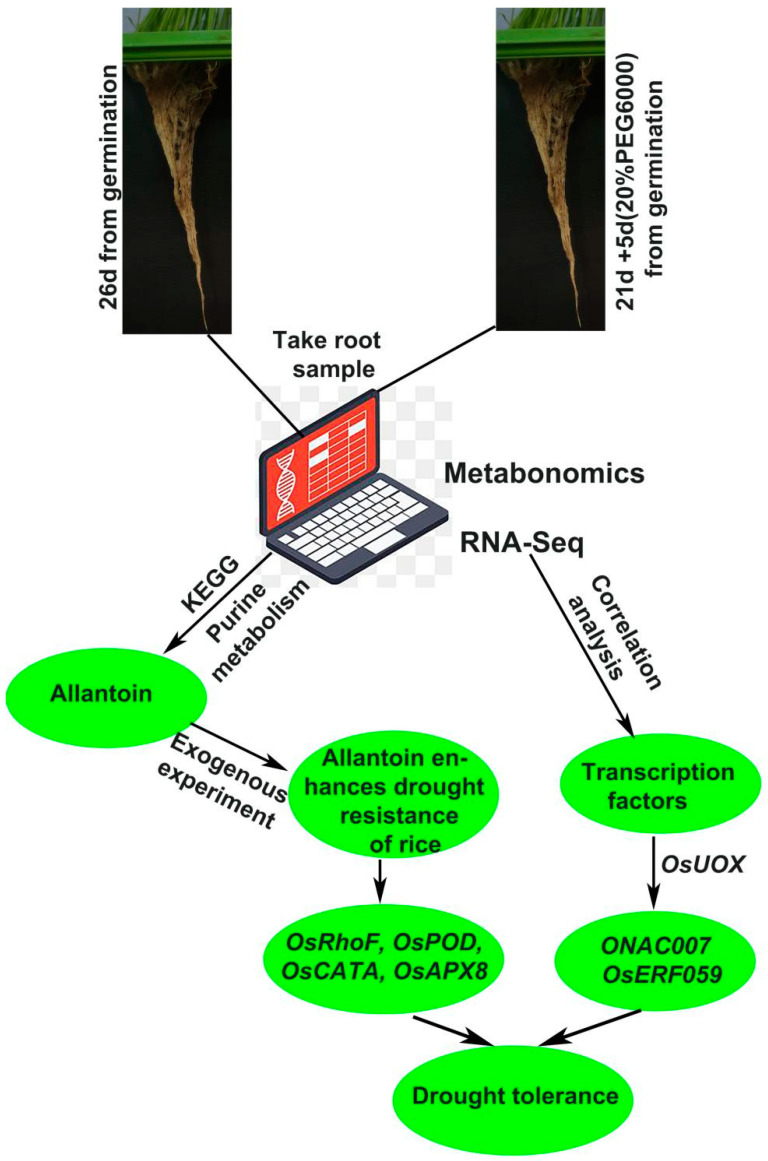
Flow chart of exogenous allantoin improving drought resistance in rice. The arrow points from one level to the next, representing the order of precedence.

The current mainstream view of the research is that rice responds to drought by scavenging ROS and abscisic acid (ABA). Overexpression of ONAC066 in rice improved the tolerance to drought and oxidative stress, increased ABA sensitivity under drought stress, decreased water loss rate, increased the content of proline and soluble sugar, reduced the accumulation of reactive ROS, and upregulated the expression of stress-related genes to enhance the drought tolerance of rice [48]. Research suggests OsRbohB may play a role through the interaction of the OsRbohB-mediated ROS production and ABA signaling in drought tolerance of rice [49]. *OsUGT85E1* gene was significantly upregulated under drought stress and ABA treatment, and overexpression of *OsUGT85E1* could induce ABA accumulation, stomatal closure, enhance ROS scavenging capacity, increase proline and sugar content, and upregulate the expression of stress-related genes under drought stress to enhance the drought resistance of rice [50]. Our study showed that the proline and soluble sugar content of rice increased, the ROS content decreased (Figure 4), and the genes related to ROS clearance were significantly upregulated under exogenous allantoin treatment (Figure 5b). This indicates that applying allantoin affects the internal ROS scavenging system of rice. Summarizing previous studies, we propose the hypothesis that exogenous allantoin may participate in the ABA pathway in response to drought, but it still needs further verification. Although similar studies in rice are rarely reported, studies in other species give us certain instructions. Growing ABA-insensitive (abi) mutants on allantoin-containing media and comparison between abi mutants and their wild-type *Arabidopsis thaliana* backgrounds demonstrated that the potential regulatory function of allantoin does not require ABA at germination but may be ABA-dependent at later stages of seedling growth, suggesting a potential crosstalk between the allantoin-mediated stress response and ABA signaling pathway in plants [51]. Research suggested that physiological changes in allantoin-treated grape berries occurred in an ABA-dependent manner. Allantoin produced bioactive ABA through the β-glucosidase-catalyzed hydrolysis of glucose-conjugated ABA but not through the ABA biosynthesis by NCED, a key enzyme in ABA biosynthesis, in berry skins. Allantoin application in viticulture under global warming conditions is expected to contribute to mitigating loss of red/black berry skin color [52]. A detailed re-examination of the microarray data of an aln mutant (*aln-1*) confirmed the increased expression of ABA-related genes and also revealed altered expression of genes involved in jasmonic acid (JA) responses, probably under the control of MYC2, a master switch in the JA signaling pathway. Consistent with the transcriptome profiles, the *aln-1* mutant displayed increased JA levels and enhanced responses to mechanical wounding and exogenous JA [53]. These studies show that allantoin participates in the ABA pathway and activates the JA pathway through the ABA pathway, which may be the key for exogenous allantoin to improve the drought resistance of rice. Allantoin can stimulate plant growth, and the ureide-degradation mutants (*aln* and *aah*) showed similar symptoms with nitrogen deficiency: early flowering, reduced size at maturity, and decreased fertility. Consistent with these phenotypes, carbon-to-nitrogen ratios and nitrogen-use efficiencies were significantly decreased in ureide-degradation mutants; however, adding nitrogen to irrigation water did not alleviate the growth decline of these mutants [54]. In addition, allantoin is also widely used in the cultivation of vegetable crops. For example, allantoin is an important regulator of sugar beet tolerance to salt stress, which is closely related to the yield of sugar beet [55]. Allantoin, as a growth regulator of urea plants, has great potential. In the increasingly changeable climate environment in the future, it is possible to achieve a win–win effect of resistance to adversity and increase production. Through the combined analysis of metabolome and transcriptome, important small molecular substances of purine metabolism pathway were found, showing exogenous allantoin can enhance the drought resistance of rice, and ROS scavenging genes (*OsRhoF*, *OsPOD*, *OsCATA* and *OsAPX8*) were upregulated. Two transcription factors (*ONAC007* and *OsERF059*) may initiate the transcription of the key gene *OsUOX* for the synthesis of allantoin (Figure 9) by correlation analysis. Allantoin plays an important role in drought resistance and crop yield improvement. The development of allantoin as one of the sources of nitrogen may solve the problem of high yield and high resistance, and make a significant contribution to food security.

When plants face drought for a long time, they will enter a mode similar to dormancy to reduce their consumption and wait for the opportunity to recover [56]. When the conditions are suitable, plants activate themselves through small molecules in the body for the first time, which is called a non-enzymatic reaction, and restore the activity at the gene level. This is an enzymatic reaction, and then restores growth [57]. The role of all metabolites and genes under drought stress and how to play a role are the key to our understanding of drought resistance in drought-tolerant plants. Although our research focuses on the allantoin metabolic pathway, and the role of metabolites and genes, there are still some interesting places that can provide guidance for us to further understand plant drought resistance. For example, in the KEGG enrichment analysis of DEGs and metabolites, they were jointly enriched into the arginine and proline metabolism pathway (Figure 2a,b). Proline is a multifunctional amino acid, which is accumulated in high concentrations in plants under various stress conditions. Proline accumulation is intimately connected to many cellular processes, such as osmotic pressure, energy status, nutrient availability, changes in redox balance, and defenses against pathogens. Proline biosynthesis and catabolism are linked to photosynthesis and mitochondrial respiration, respectively. Proline can function as a signal, modulating gene expression and certain metabolic processes [58]. DEGs were also enriched in glycolysis/glucogenesis, while DEMs were enriched in the pentose phosphate pathway and citrate cycle (TCA cycle). They are all related to energy metabolism, which may be the key for plants to enter dormancy or break dormancy and recover through genes or metabolites. This requires further research. However, when plants face drought, the metabolic pathways in which plants participate are complex and diverse and may be connected in series. Polyamine metabolism interacts with γ- aminobutyric acid, proline, and nitrogen metabolisms to affect drought tolerance of *Creeping Bentgrass* [59]. In our study, nitrogen metabolism, purine metabolism, pyrimidine metabolism, glutathione metabolism, isoquinoline alkaloid biosynthesis, and other amino acid metabolism pathways are enriched, which are all related to nitrogen metabolism. What kind of connection they have and which way rice dominates in the face of drought stress are worth exploring in depth.

## 4. Materials and Methods

### 4.1. Plant Materials and Growth Conditions

Rice Wild-Type (WT), Nipponbare was selected in this experiment and cultivated in soil and water. Seeds were germinated and grown in vermiculite and nutrient soil (*v*:*v* = 1:3) in an artificial climate chamber (16 h light, 8 h darkness, 23 °C). Soil volume in each pot was the same before germination. After 10 days of germination, the number of seedlings in each pot was artificially controlled to be unified, and the plants were grown further in the same manner. All plants grew under the same water conditions until 3-weeks-old, watering was withheld and photographs were obtained after 2 weeks to record the drought phenotype. Under hydroponic culture conditions, the rice seeds were soaked and germinated in clean water. After 10 days, the Kimura B nutrient salt solution was changed every 3 days. Allantoin treatment is to add allantoin to the nutrient solution to form allantoin nutrient solution of corresponding concentration. Drought was simulated with 20% PEG6000 (*w*:*v*) for 3-week-old rice seedlings. After 5 days of PEG treatment, the roots and leaves were collected and stored at −80 °C for sequencing and subsequent experiments, measuring physiological indices, respectively.

### 4.2. RNA-Seq and Data Analysis

The transcriptome of NIP roots under normal (NIP-CK) and drought (NIP-D) conditions was sequenced by PANOMIX Biomedical Tech Co., Ltd. (Suzhou, China). There were two treatments, with three independent replicates per treatment, for 6 samples in total. The original RNA-seq data were submitted to the National Genomics Data Center (Bioproject: PRJCA012202). Quality control was confirmed using the Illumina HiSeq software (Illumina, San Diego, CA, USA), and all readings that passed the filter specifications were plotted on the reference genome IRGSP-1.0. Using the R package DESeq (Panomix, Suzhou, China), PCA was performed on each sample according to the expression. After calculating the expression level of each transcript and gene, DESeq was used for differential expression analysis. The conditions for screening DEGs were as follows: expression difference in multiple |log2 foldchange| > 1 and significance *p* value < 0.05. A volcano map of DEGs was drawn using the R package ggplot2. KEGG and GO enrichment analyses were performed using OmicShare [60] (www.omicshare.com/tools, accessed on 16 June 2022). Related differential alternative splicing and splicing factor analysis was performed according to Chen et al. [61].

### 4.3. Metabolomic Analysis

#### 4.3.1. Extraction Method of Metabolites

PANOMIX Biomedical Tech Co., Ltd. (Suzhou, China) performed non-targeted metabolomics. There were two treatments, with six independent replicates per treatment, 12 samples in total. The reagents and methods used for extracting metabolites were carried out as described by Vasilev et al. [62] (2016). Liquid chromatography (LC) was performed using the Vanquish UHPLC System (Thermo Fisher Scientific, Waltham, MA, USA). Chromatography was performed using ACQUITY UPLC ^®^ HSS T3 (150 mm × 2.1 mm, 1.8 µm) (Waters, Milford, MA, USA).

#### 4.3.2. Sample Online Detection

The column was maintained at 40 °C. The flow rate and injection volume were set at 0.25 mL/min and 2 μL, respectively. For LC-ESI (+)-MS analysis, the mobile phases consisted of 0.1% formic acid in acetonitrile (*v*/*v*) and 0.1% formic acid in water (*v*/*v*). For LC-ESI (-)-MS analysis, the analytes were carried out with acetonitrile and ammonium formate (5 mM) [63]. Gradient elution was performed following the method described by Zelena et al. [63]. Mass spectrometric (MS) detection of metabolites was performed using Orbitrap Exploris 120 with an ESI ion source (Thermo Fisher Scientific, Waltham, MA, USA). The parameters for positive and negative ion modes were determined as described by Want (2013) [64].

#### 4.3.3. Data Processing and Analysis

The raw data (Bioproject: PRJCA012202) were firstly converted to mzXML format by MSConvert in ProteoWizard software package (v3.0.8789) [65] and processed using XCMS [66] for feature detection, retention time correction and alignment. The metabolites were identified by accuracy mass (<30 ppm) and MS/MS data, which were matched with HMDB [67] (http://www.hmdb.ca, accessed on 16 March 2022), massbank [68] (http://www.massbank.jp/, accessed on 16 March 2022), LipidMaps [69] (http://www.lipidmaps.org, accessed on 16 March 2022), mzclound [70] (https://www.mzcloud.org, accessed on 16 March 2022) and KEGG [71] (http://www.genome.jp/kegg/, accessed on 16 March 2022). The robust LOESS signal correction (QC-RLSC) [72] was applied for data normalization to correct for any systematic bias. After normalization, only ion peaks with relative standard deviations (RSDs) less than 30% in QC were kept to ensure proper metabolite identification. OPLS-DA allowed the determination of discriminating metabolites using the variable importance on projection (VIP). The *p* value, VIP produced by OPLS-DA, fold change (FC) was applied to discover the contributable-variable for classification. Finally, *p* value < 0.05 and VIP values > 1 were considered to be statistically significant metabolites. Differential metabolites were subjected to pathway analysis by MetaboAnalyst [73], which combines results from powerful pathway enrichment analysis with the pathway topology analysis. The identified metabolites in metabolomics were then mapped to the KEGG pathway for biological interpretation of higher-level systemic functions. The metabolites and corresponding pathways were visualized using KEGG Mapper (http://www.kegg.jp/, accessed on 18 March 2022). Transcriptome and metabolome association and correlation network analyses were performed using OmicShare (https://www.omicshare.com, accessed on 16 June 2022).

### 4.4. qRT-PCR Analyses of Gene Expression

Plant RNA extraction was performed according to the instructions of the RNA simple Total RNA Kit from TIANGEN. cDNA synthesis was performed according to the instructions of the FastKing one-step genomic cDNA first strand synthesis premix reagent from TIANGEN. Fluorescent quantitative PCR was performed using the previously synthesized cDNA as a template by following the instructions provided with the FS Universal SYBR Green Master Kit from ROCHE. *OsACTIN-1* (*LOC_Os05g36290*) was used as the internal reference to detect the expression level of each target gene, and three or more replicates were set for each sample and the internal reference. The relative primer sequences of all detected genes were showed in Table S2. The relative expression levels genes were calculated according to the method [74].

### 4.5. Physiological Measurements

The soluble sugar content was determined using the anthrone method [75]. Briefly, the leaves of 3-week-old plants treated with 20% PEG6000 for 5 days were collected. Leaf tissues (0.5 g) were extracted in 15 mL of distilled water by boiling for 20 min with constant stirring. The supernatant was filtered and raised to a final volume of 100 mL with distilled water. Then, 1 mL of the extract was incubated with 5 mL of anthrone reagent at 95 °C for 15 min. The reaction was terminated on ice, followed by the measurement of the absorbance at 620 nm. ROS content was measured using the fluorescent probe H2DCFD (medchemexpress, Monmouth Junction, NJ, USA) method in accordance with the manufacturer’s instructions. Pro content was analyzed using commercial kits (Nanjing Jiancheng Bioengineering Institute, Nanjing, China). Contents were detected with a microplate detector (EnSpire (TM) 2300, Waltham, MA, USA) and an ultraviolet spectrophotometer (Evolution 300, Waltham, MA, USA).

### 4.6. Promoter Motif Prediction

The 2.0-kb promoter sequences of plant *OsUOX* genes were extracted from the Phytozome database and used for the prediction of cis-elements using PlantCARE (http://bioinformatics.psb.ugent.be/webtools/plantcare/html/, accessed on 16 September 2022) [76].

### 4.7. Statistical Analysis

SPSS v.25.0 (Chicago, IL, USA) and GraphPad Prism v.9.0.0 (Chicago, IL, USA) were used for statistical analysis and charting. Significant differences in the figures follows the rules: **** *p* < 0.0001, *** *p* < 0.001, ** *p* < 0.01, and * *p* < 0.05.

## 5. Conclusions

In this study, we found that allantoin metabolism in purine metabolism may play an important role in drought stress through transcriptome and metabolome analysis. Under the treatment of different concentrations of exogenous allantoin, the drought-resistant metabolites of plants accumulated significantly, including proline and soluble sugar, and ROS decreased. Exogenous allantoin can improve the drought resistance of rice seedlings. At the gene level, we found that genes for scavenging reactive oxygen species were significantly expressed, including peroxidase (POD), catalase (CATA), ascorbate peroxidase 8 (APX8), and respiratory burst oxidase homolog protein F (RbohF) under allantoin drought treatment. This indicates that allantoin-treated plants have a better ability to scavenge reactive oxygen species to resist drought. Through correlation analysis, we found OsERF059 and ONAC007 transcription factors highly related to allantoin accumulation, which pointed out the direction for our follow-up work. In addition, based on the development of SWATH-MS proteomics research in recent years, it provides technical support for the in-depth follow-up related research.

## Data Availability

The original RNA-seq data or metabolome data have been deposited in National Genomics Data Center, China National Center Bioinformation/Beijing Institute of Genomics, Chinese Academy of Sciences (BioProject: PRJCA012202).

## Supplementary Materials

The supporting information can be downloaded at: https://www.mdpi.com/article/10.3390/ijms232214172/s1.
