# Supplementary figures and images for: Combined Metabolomic and Transcriptomic Analysis Reveals Allantoin Enhances Drought Tolerance in Rice
Source: Int J Mol Sci. 2022 Nov 16;23(22):14172. doi: 10.3390/ijms232214172 (PMC9699107; doi:10.3390/ijms232214172)

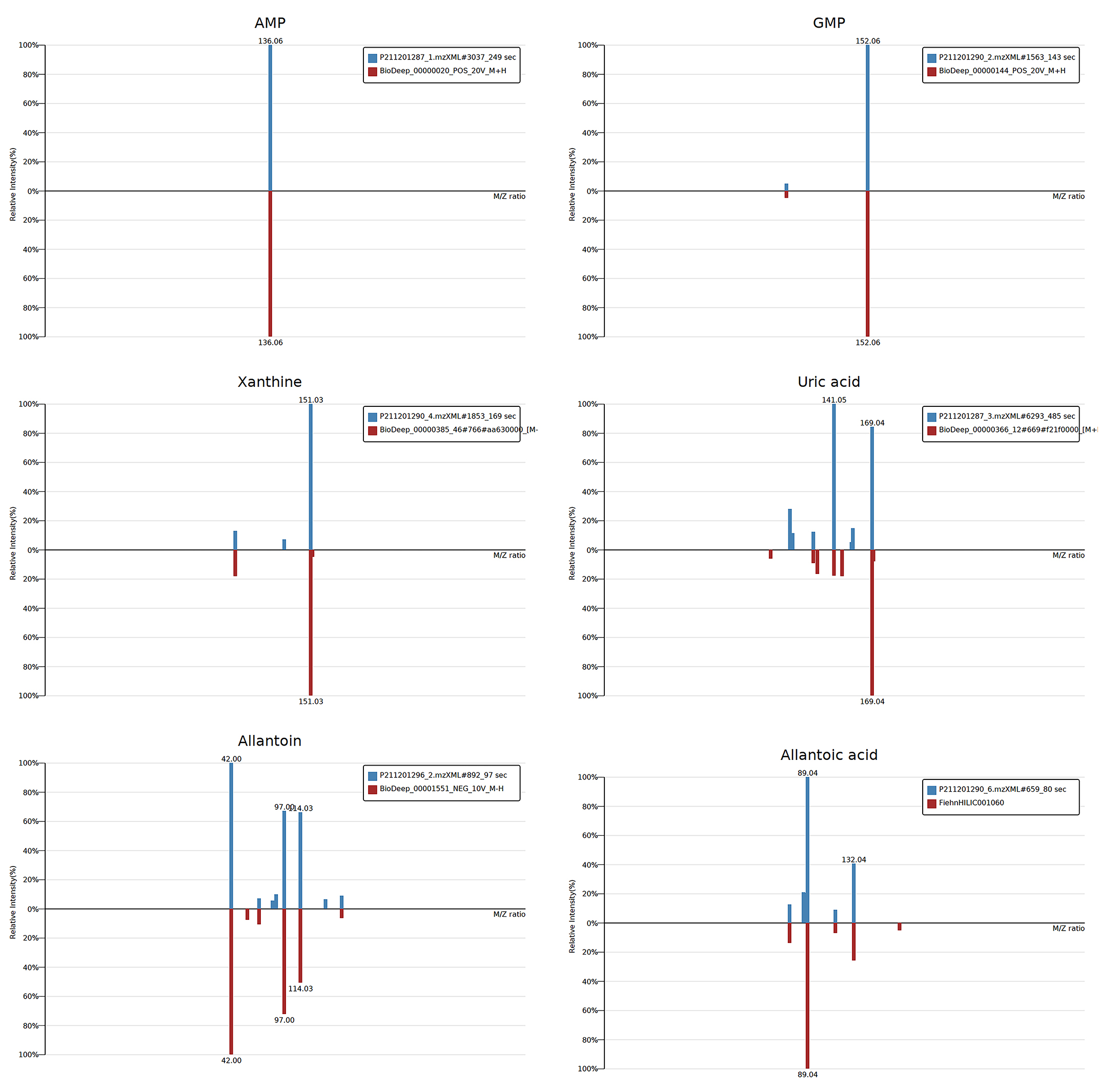

Supplement: Supplementary file 1 [file ijms-23-14172-s001.zip › Figure S1.jpg]

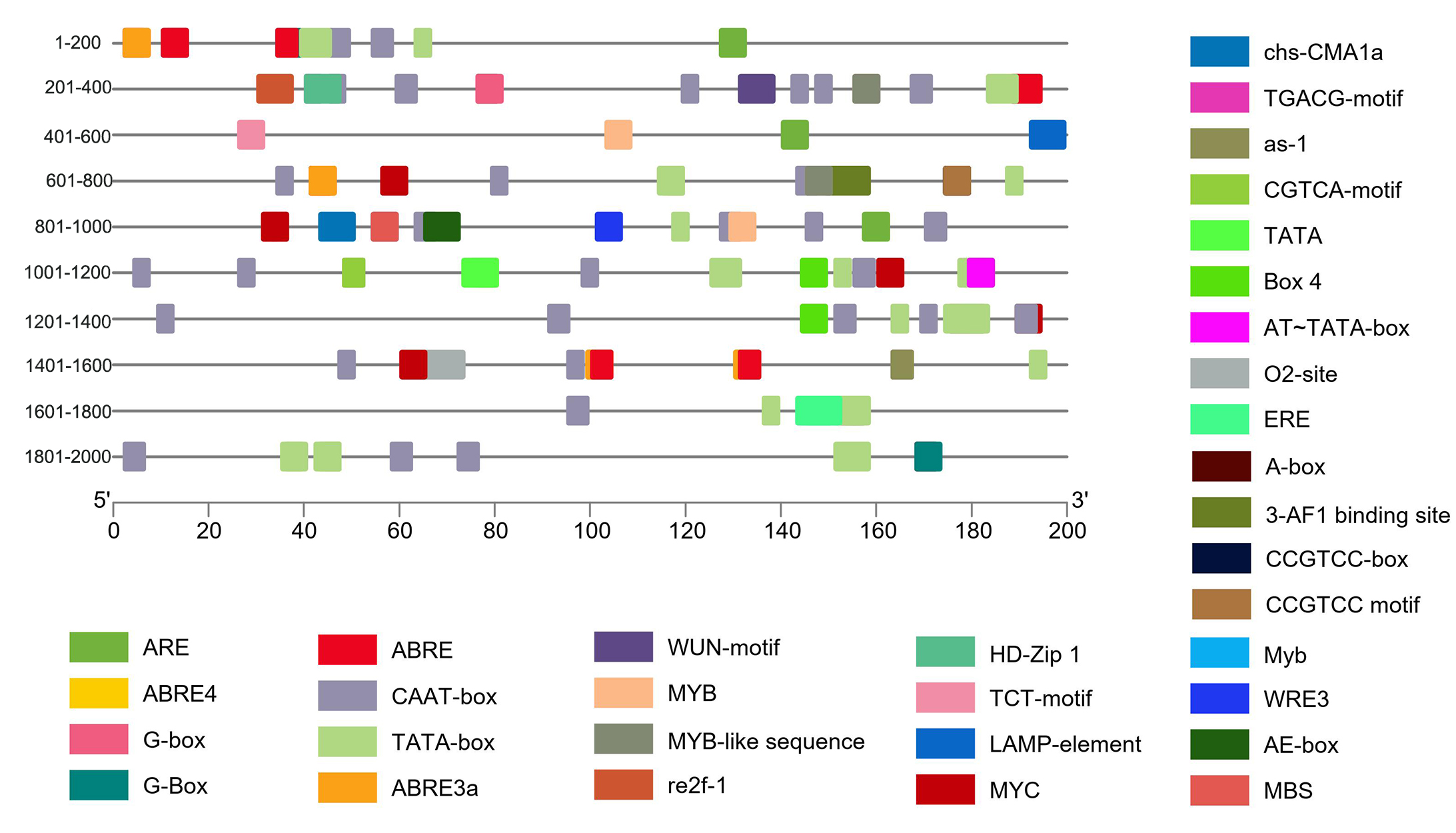

Supplement: Supplementary file 1 [file ijms-23-14172-s001.zip › Figure S2.jpg]
